# Supplementary material for: The interactome of intact mitochondria by cross-linking mass spectrometry provides evidence for coexisting respiratory supercomplexes
Source: Mol Cell Proteomics. 2017 Dec 8;17(2):216–32. doi: 10.1074/mcp.RA117.000470 (PMC5795388; doi:10.1074/mcp.RA117.000470)
Supplement: Supplemental Data [file supp_RA117.000470_133922_0_supp_23978_fzffwf.zip › spectra_annotation/mito_DR_spectra_annotation/220-1-5-1-7-1.pdf]

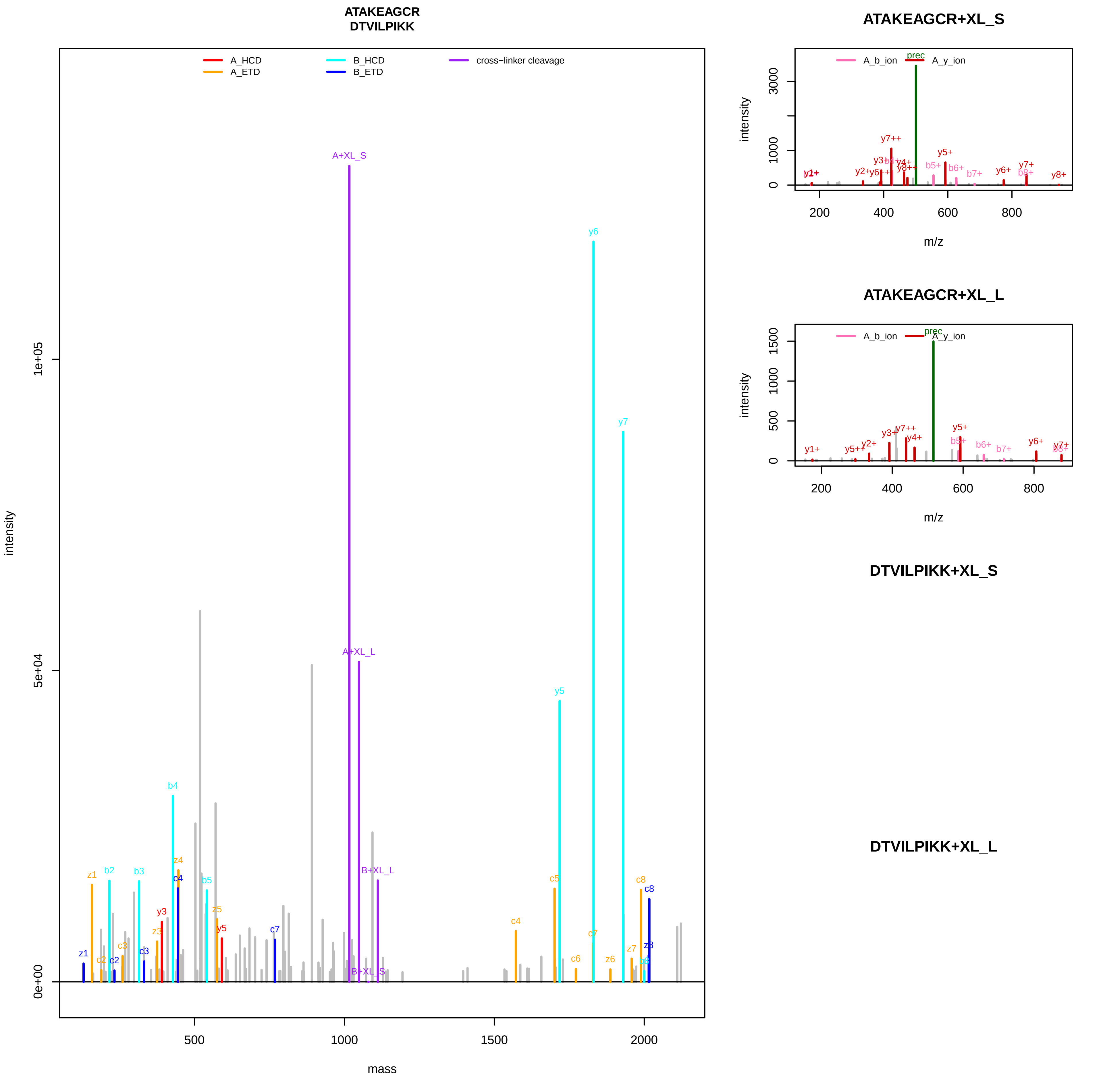
ATAKEAGCR
DTVILPIKK
A_HCD
A_ETD
B_HCD
B_ETD
cross−linker cleavage
A+XL_S
A+XL_L
B+XL_L
B+XL_S
y6
y7
y5
b4
b2
b3
b5
z1
z4
c4
c5
c8
y3
y5
z5
c7
c4
c6
z6
z7
c2
c3
z2
z3
z8
b8
intensity
mass
0e+00
5e+04
1e+05
500
1000
1500
2000
ATAKEAGCR+XL_S
A_b_ion
A_y_ion
prec
intensity
m/z
0
1000
3000
200
400
600
800
y7++
y5+
y1+
y2+
y3+
y4+
y7+
y8+
y6+
b5+
b6+
b7+
b8+
ATAKEAGCR+XL_L
A_b_ion
A_y_ion
prec
intensity
m/z
0
500
1000
1500
200
400
600
800
y1+
y5++
y2+
y3+
y7++
y4+
y5+
b5+
b6+
b7+
y6+
y7+
b8+
DTVILPIKK+XL_S
DTVILPIKK+XL_L
